# Editorial for the Special Issue on Recent Advances in Reactive Ion Etching and Applications of High-Aspect-Ratio Microfabrication

**DOI:** 10.3390/mi14081630

**Published:** 2023-08-18

**Authors:** Lucia Romano, Konstantins Jefimovs

**Affiliations:** 1 Institute for Biomedical Engineering, University and ETH Zürich, 8092 Zürich, Switzerland; 2 Paul Scherrer Institute, 5232 Villigen, Switzerland

Reactive ion etching (RIE) is the dominating technology for micromachining semiconductors with a high aspect ratio (HAR). It has long been recognized as a powerful manufacturing tool and has been widely applied across different research fields and industrial sectors. The semiconductor industry is continuously pushing to shrink lateral dimensions and to increase the aspect ratio of features while maintaining a cost advantage. This is driving new challenges in RIE processes to meet the demands of control feature-size variation and fabrication of HAR features in high-volume manufacturing. Recent developments in RIE methods and equipment have enabled considerably deeper etches and higher aspect ratios compared to traditional RIE methods and have revolutionized bulk micromachining technologies. The most widely known of these technologies is called inductively coupled plasma (ICP) deep reactive ion etching (DRIE), and this has become a mainstay for the development and production of silicon-based micro- and nano-machined devices. In the ICP DRIE systems, the plasma is generated with an RF-powered magnetic field, and a separate RF generator directs an electrical field to steer the reactants toward the substrate and obtain a highly anisotropic etch result [1]. ICP has been widely used as a plasma source because of its advantages, such as simple equipment configuration and high-density plasma at a low pressure. The Bosch process is currently one of the key processes in the silicon industry. There is a standard sequence of basic operations used to fabricate the required device topology on a silicon substrate, which are surface preparation, photoresist spin coating, photolithography, photoresist development, mask pattern transfer, photoresist removal, and silicon etching [2]. This Special Issue addresses new challenges and updates the state of the art about deep reactive ion etching.

On one side, several papers address typical problems of pattern transfer, such as the mask effect on the lateral undercut [3] and etch lag [4]. Different techniques, such as Bosch and STiGer, are compared [5] and reviewed [1] in order to provide a wider overview of their new capabilities. New plasma sources such as ICP systems using burst waves are explored to increase the silicon etching rate [6]. Advanced microsystems that are widely used in integrated optoelectronic devices, energy harvesting components, and microfluidic lab-on-chips require high-aspect silicon microstructures with a precisely controlled profile. Such microstructures can be fabricated using the Bosch process, which is a key process for the mass production of micro-electro-mechanical systems (MEMS) devices, where DRIE can also be used to improve the metrology in controlled cleaving for cross sections [2]. On the other side, the research efforts regarding optical devices are focused on bringing non-conventional micro- and nanofabrication into established silicon processing lines. In many research fields of process engineering technical innovations are triggered by the applications. In this Special Issue, the recent advances of DRIE in new emerging sectors of optics, such as InP/InGaAsP heterostructures [7], Si [8] and Si/Au gratings [9] for X-ray imaging, and structured nanopillars for metasurfaces [10], are reported.
